# Mapping research evidence on perinatal asphyxia within the sustainable development era in sub-Saharan Africa: a scoping review protocol

**DOI:** 10.1186/s13643-022-02058-4

**Published:** 2022-08-30

**Authors:** Michael K. Mensah, Franky K. Tano Kofi, Monica Ansu-Mensah, Vitalis Bawontuo, Desmond Kuupiel

**Affiliations:** 1 Department of Public Health, Faculty of Health and Allied Sciences, Catholic University, College of Ghana, Fiapre, Sunyani, Ghana; 2 The University Clinic, Sunyani Technical University, Sunyani, Ghana; 3 Department of Health Services Management and Administration, School of Business, SD Dombo University of Business and Integrated Development Studies (SDD-UBIDS), Bamahu, Ghana; 4 Faculty of Health Sciences, Durban University of Technology, Durban, 4001 South Africa

**Keywords:** Perinatal asphyxia, Birth asphyxia, Neonate, Newborn, Sustainable Development Goal, Africa, Scoping review

## Abstract

**Background:**

Approximately 45% of all under-five child deaths are among newborn infants, babies in their first 28 days of life, or the neonatal period every year in the World Health Organization (WHO) Africa Region. To facilitate the achievement of Sustainable Development Goal (SDG) 3.2, innovative interventions are needed to address this challenge. Thus, this scoping review aims to map research evidence on perinatal asphyxia among neonates in the WHO Africa Region.

**Methods:**

This scoping review will be guided by the Arksey and O’Malley framework, Levac et al. recommendations, and the Joanna Briggs Institute checklist for scoping reviews. Relevant published literature will be searched using a combination of keywords, Boolean terms, and Medical Subject Headings in the following databases: PubMed, SCOPUS, CINAHL, and Web of Science from January 2016 onwards. We will further search the World Health Organization and government websites, as well as the reference list of included studies for potentially eligible studies. This scoping review will include research evidence involving countries in the WHO Africa Region, with a focus on the burden of perinatal asphyxia, contributory/associated factors of perinatal asphyxia, clinical interventions for perinatal asphyxia, and interventions/strategies for the prevention of perinatal asphyxia in the SDGs era. Two reviewers will independently sort the studies to include and exclude, guided by the eligibility criteria. Also, the data from the included studies will be extracted and any discrepancies resolved using a third reviewer. Thematic analysis will be conducted, and the findings reported using both qualitative tables and figures. The Preferred Reporting Items for Systematic Reviews and Meta-Analysis Extension for Scoping Review (PRISMA-ScR) will be followed to report this study’s results. Quality appraisal of the included studies will be done utilising the mixed methods appraisal tool version 2018.

**Conclusion:**

This scoping review results may reveal research evidence gaps to inform future primary studies, systematic reviews and meta-analyses; and possibly contribute towards the realisation of the SDG 3.2 by countries in the WHO Africa Region. The finding of this review will be disseminated using multiple channels such as workshops, peer review publications, conferences, and social media.

**Supplementary Information:**

The online version contains supplementary material available at 10.1186/s13643-022-02058-4.

## Background

Perinatal asphyxia is a lack of blood flow or gas exchange to or from the foetus in the period immediately before, during, or after the birth process [1]. In other words, the World Health Organization [2] defined perinatal asphyxia as “the failure to initiate and sustain breathing at birth” [2]. A diagnosis of asphyxia is established, when a newborn has an APGAR (Appearance, Pulse, Grimace, Activity, and Respiration) score of less than seven at the first to the fifth minute after birth. Birth asphyxia results in impairment of tissue perfusion leading to hypoxemia and metabolic acidosis with multi-organ failure or death [3]. Globally, an estimated 4 million newborns die in the neonatal period; 3 million of them died within 7 days of life [3]. More than 99% of neonatal mortality occurs in lower-and-middle-income countries (LMICs) [4]. Neonatal mortality accounted for 46% of under-five mortality in 2014 and it is estimated to increase to 52% by 2030 if appropriate measures are not implemented to address it [5]. Moreover, perinatal asphyxia is said to be responsible for about 42 million disability-adjusted life years (DALYs) [6]. To this end, reducing preventable newborn deaths is part of the global health agenda captured in the Sustainable Development Goals (SDGs) [7].

The United Nations Sustainable Development Goal three (SDG 3) seeks among other targets to end preventable deaths of newborns and children under 5 years of age, with all countries aiming to reduce neonatal mortality to less than 12 per 1000 live births and under-5 mortality to less than 25 per 1000 live births by 2030 (SDG 3.2) [7]. In LMICs however, the proportion of birth asphyxia is estimated to be more than ten (10) times higher compared to high-income countries (HIC) (2 per 1000 births in HIC) [8]. To reduce neonatal mortality to less than 12 per 1000 live births, interventions such as improving skills of birth attendance, increasing the availability of emergency obstetric care, and training of this personnel with access to resuscitation equipment among others have been suggested [9, 10]. However, there is the need to understand the contributory factors to enable the scale-up or implementation of appropriate clinical and contextual preventive interventions. Thus, to reduce newborn deaths and DALYs attributable to perinatal asphyxia, research evidence on burden of perinatal asphyxia, contributory/associated factors of perinatal asphyxia, clinical interventions for perinatal asphyxia, and interventions/strategies for the prevention (primary, secondary and tertiary) [11] of perinatal asphyxia is crucial.

Although several primary studies and systematic reviews may have been conducted in the past relating to perinatal asphyxia, about 45% of all under-five child deaths are among newborn infants, babies in their first 28 days of life or the neonatal period every year in the WHO Africa Region [12]. To this end, it is essential to identify literature glut and gaps in order to guide subsequent research and health policy decisions and reforms. Therefore, this study aims to systematically map and describe the range of evidence relating to perinatal asphyxia in the SDGs era in the WHO Africa Region.

## Methods

This scoping review will be conducted using the Arksey and O’Malley framework (identifying the research question; identifying relevant studies; study selection; charting the data; and collating, summarising and reporting results) [13] including the Levac et. al. recommendations [14]. Also, the recent Joanna Briggs Institute guidelines for scoping reviews will be consulted [15]. The Preferred Reporting Items for Systematic and Meta-Analyses Checklist was used as a guide to write this protocol (Supplementary file 1).

### Identifying the research question

This scoping review study’s question will be: For children less than 28 days old, what research evidence on perinatal asphyxia exists in the WHO Africa Region within the SDGs era? The sub-review questions will be as follows:

- What research evidence exists on the burden (incidence, prevalence, and mortality) of perinatal asphyxia in the WHO Africa Region?
- What research evidence exists on the contributory/associated factors of perinatal asphyxia in the WHO Africa Region?
- What research evidence exists on interventions/strategies for the prevention (primary, secondary, and tertiary) of perinatal asphyxia in the WHO Africa Region?
- What research evidence exists on clinical interventions for perinatal asphyxia in the WHO Africa Region?

The eligibility criteria for this review are defined in Table 1 below.

**Table 1:** Eligibility criteria of studies for this review

Criterion	Include	Exclude
Population	Neonates or newborns with the first 4 weeks of a child’s life (28 days)	
Concept	Perinatal asphyxia	
Setting	All Countries within the WHO Africa Region	Other WHO Regions
Study designs	Quantitative, qualitative, mix-method study designs as well as systematic review, and/or meta-analyses/meta-synthesis	Editorials, comments, expert opinions, and others
Time frame	From 2016 onwards (SDGs era)	Studies published prior to Jan 1 2016
Language	All publication languages	

### Identify relevant studies

Relevant published literature that meets this review criteria will be sought in PubMed, SCOPUS and CINAHL, and Web of Science to answer the review question. The search engine Google Scholar and the World Health Organization and government websites, and the reference list of included studies will be searched to identify potentially eligible studies. The electronic search strategy will be developed in consultation with an expert librarian and guided by the Peer Review of Electronic Search Strategies (PRESS) statement. The search based on an expert librarian’s advice will comprise of keywords, Boolean terms, and Medical Subject Heading (MeSH) terms or Subject Headings where applicable (Table 2). Based on the database, the syntax will be modified. During the database search, language and study design restrictions will be removed, but the date (from 2016 onwards to the last search date) will be restricted. The Principal Investigator (MKM) will conduct the search with the help of the expert librarian. The search results will be documented and imported onto Mendeley Desktop Library.

**Table 2:** A pilot search in PubMed electronic database for this scoping review

Date	Database	Search No.	Query	Search result
26/05/2022	PubMed	# 1	"infant"[MeSH Terms] OR "infant"[All Fields] OR "infants"[All Fields] OR "infant s"[All Fields] OR ("infant, newborn"[MeSH Terms] OR ("infant"[All Fields] AND "newborn"[All Fields]) OR "newborn infant"[All Fields] OR "newborn"[All Fields] OR "newborns"[All Fields] OR "newborn s"[All Fields]) OR ("infant, newborn"[MeSH Terms] OR ("infant"[All Fields] AND "newborn"[All Fields]) OR "newborn infant"[All Fields] OR "baby"[All Fields] OR "infant"[MeSH Terms] OR "infant"[All Fields]) OR ("infant, newborn"[MeSH Terms] OR ("infant"[All Fields] AND "newborn"[All Fields]) OR "newborn infant"[All Fields] OR "neonatal"[All Fields] OR "neonate"[All Fields] OR "neonates"[All Fields] OR "neonatality"[All Fields] OR "neonatals"[All Fields] OR "neonate s"[All Fields]) OR ("child"[MeSH Terms] OR "child"[All Fields] OR "children"[All Fields] OR "child s"[All Fields] OR "children s"[All Fields] OR "childrens"[All Fields] OR "childs"[All Fields]) OR ("paediatrics"[All Fields] OR "pediatrics"[MeSH Terms] OR "pediatrics"[All Fields] OR "paediatric"[All Fields] OR "pediatric"[All Fields]) OR ("paediatrics"[All Fields] OR "pediatrics"[MeSH Terms] OR "pediatrics"[All Fields] OR "paediatric"[All Fields] OR "pediatric"[All Fields]) OR ("infant"[MeSH Terms] OR "infant"[All Fields] OR "infants"[All Fields] OR "infant s"[All Fields]) OR ("infant, newborn"[MeSH Terms] OR ("infant"[All Fields] AND "newborn"[All Fields]) OR "newborn infant"[All Fields] OR "newborn"[All Fields] OR "newborns"[All Fields] OR "newborn s"[All Fields]) OR ("baby s"[All Fields] OR "babys"[All Fields] OR "infant"[MeSH Terms] OR "infant"[All Fields] OR "babies"[All Fields]) OR ("infant, newborn"[MeSH Terms] OR ("infant"[All Fields] AND "newborn"[All Fields]) OR "newborn infant"[All Fields] OR "neonatal"[All Fields] OR "neonate"[All Fields] OR "neonates"[All Fields] OR "neonatality"[All Fields] OR "neonatals"[All Fields] OR "neonate s"[All Fields]) OR ("child"[MeSH Terms] OR "child"[All Fields] OR "children"[All Fields] OR "child s"[All Fields] OR "children s"[All Fields] OR "childrens"[All Fields] OR "childs"[All Fields])	3,938,298
		# 2	(("perinatal"[All Fields] OR "perinatally"[All Fields] OR "perinatals"[All Fields]) AND "asphyxia"[MeSH Terms]) OR "asphyxia neonatorum"[MeSH Terms] OR (("perinatal"[All Fields] OR "perinatally"[All Fields] OR "perinatals"[All Fields]) AND ("asphyxia"[MeSH Terms] OR "asphyxia"[All Fields] OR "asphyxias"[All Fields])) OR ("asphyxia neonatorum"[MeSH Terms] OR ("asphyxia"[All Fields] AND "neonatorum"[All Fields]) OR "asphyxia neonatorum"[All Fields] OR ("birth"[All Fields] AND "asphyxia"[All Fields]) OR "birth asphyxia"[All Fields])	12,384
		# 3	"africa south of the sahara"[MeSH Terms] OR ("africa"[All Fields] AND "south"[All Fields] AND "sahara"[All Fields]) OR "africa south of the sahara"[All Fields] OR ("sub"[All Fields] AND "saharan"[All Fields] AND "africa"[All Fields]) OR "sub saharan africa"[All Fields] OR "SSA"[All Fields]	261,558
		# 4	# 1 AND #2 AND # 3	502

### Study selection

**Fig. 1:**
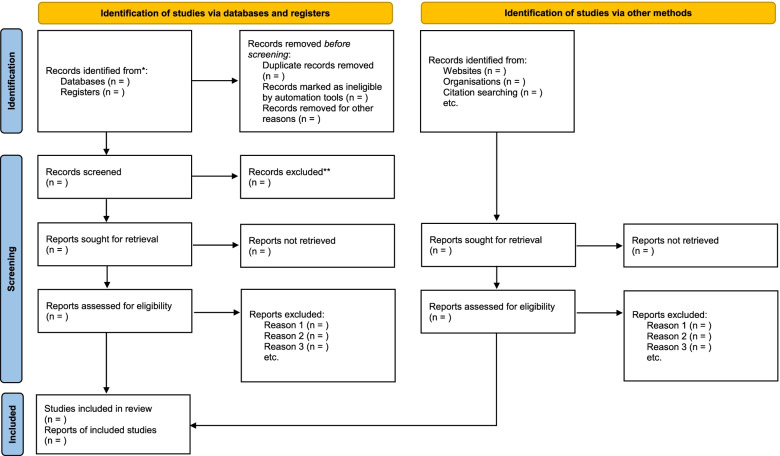
PRISMA 2020 flow diagram

The selection of the studies will be conducted in two phases using pretested screening forms. At the first phase (titles and abstracts screening), duplicate articles will be removed, and two reviewers (MKM and FKT) will independently apply the review eligibility criteria to include articles that meet the criteria and exclude those not eligible. Any disagreement in this phase will be resolved through discussions. At the second phase, the full-text articles will be compiled and screened by two independent reviewers (MKM and FKT) using the eligibility criteria. Discrepancies between reviewers’ responses at this phase will be addressed using a third reviewer (DK). In the situation where a full-text article cannot be found from the databases, assistance would be sought from the Catholic University’s library and the Stellenbosch University Library or request the full text from the author(s) via their email address (es) for screening. The PRISMA 2020 flow diagram (Fig. 1) will be used to document the study selection process [16].

### Charting the data

A data charting form will be developed and piloted using at least 10% of the included studies by two independent reviewers. Differences of opinion will be resolved through discussion and the data extraction form modified accordingly. The author and date, study title, study objective/aim, study design, study location (country), study setting (facility-based, community-based), geographical location (rural and urban), study findings (incidence, prevalence, outcomes, contributory/associated factors, clinical interventions, and preventive interventions) will be extracted from the included studies independently by two reviewers (MKM and FKT). A third reviewer (DK) will resolve any discrepancies.

### Quality appraisal

Mixed Method Quality Appraisal Tool (MMAT) Version 2018 [17] will be used to evaluate the methodological quality of all the studies included in this proposed scoping review**.** The relevance of the study, study design, adequacy and methodology, data collection, analysis of data, and study findings reported of all included will be examined using the MMAT tool. Quality assessment will be helpful in reporting the risk of bias of the studies included. The quality of the incorporated studies will be categorised by crafting the entire percentage quality score as specified by the 2018 MMAT. A percentage quality score ranging from ≤50% will be considered as low quality, 51–75% will be examined as average quality, and 76–100% will be considered as high quality.

### Collating, summarising, and reporting the results

The analysis of the data collated will include descriptive analysis to describe the characteristics of the included studies and present using frequencies (percentage), tables, figures and maps. However, qualitative synthesis of the study findings through thematic analysis. Based on initial coding and categorisation, a thematic analysis will be utilised to define the themes linked with this study's research questions. A summary of the study findings will be reported qualitatively for each theme. All emerging sub-themes relating to perinatal asphyxia will be structured around the following: burden of perinatal asphyxia, contributory/associated factors of perinatal asphyxia, clinical interventions for perinatal asphyxia, interventions/strategies for the prevention of perinatal asphyxia in the WHO Africa Region. This study will not undertake a meta-analysis due to the exploratory nature of scoping review studies; however, a follow-up meta-analysis using the quantitative data from this study may be conducted. The PRISMA extension for scoping reviews will be followed to present the results.

## Discussions

Approximately 45% of all under-five child deaths are among newborn infants, babies in their first 28 days of life or the neonatal period every year in the WHO Africa Region. Innovative interventions are needed to address this challenge. Hence, this scoping review aims to map research evidence on perinatal asphyxia among neonates in the WHO Africa Region focusing on the burden of perinatal asphyxia, contributory/associated factors of perinatal asphyxia, clinical interventions for perinatal asphyxia, interventions/strategies for the prevention of perinatal asphyxia. It is anticipated that the results of this study will lead to further research and provide evidence-based information to address perinatal asphyxia among neonates in the WHO Africa Region. This study will exclude studies conducted in the millennium development era (before 1^st^ January 2016) in order to present recent research evidence. The finding of this review will be disseminated using multiple channels such as workshops, peer review publications, conferences, and social media.

## Conclusion

This scoping review results may reveal research evidence gaps to inform future research such as primary studies, systematic reviews, and meta-analyses and possibly contribute towards the realisation of the SDG 3.2 by countries in the WHO Africa Region.

## Supplementary Information

**Additional file 1.** PRISMA-P 2015 Checklist

## Data Availability

We have duly cited all studies and data is presented in the form of references.

## Abbreviations

PCC: Population, Concept and Context
LMIC: Lower- and middle-income countries
ANC: Antenatal care
MMAT: Mixed Method Quality Appraisal Tool
SSA: Sub-Sahara African
WHO: World Health Organization
DALYs: Disability-adjusted life years
APGAR: Appearance Pulse Grimace Activity and Respiration
HIC: High-income countries
MeSH: Medical Subject Heading
SDG: Sustainable Development Goal
